# Impact of Thrombophilic Polymorphisms in Antenatal Women on Perinatal Health: A Single-Center Prospective Study

**DOI:** 10.3390/jpm14040433

**Published:** 2024-04-19

**Authors:** Vesna Sokol Karadjole, Antonio D’Amato, Milan Milošević, Mislav Herman, Mislav Mikuš, Antonio Simone Laganà, Vito Chiantera, Andrea Etrusco

**Affiliations:** 1 Department of Obstetrics and Gynecology, University Hospital Center Zagreb, 10000 Zagreb, Croatia; vskaradjole@gmail.com (V.S.K.); mislav.herman@gmail.com (M.H.); m.mikus19@gmail.com (M.M.); 2 School of Medicine Zagreb, University of Zagreb, 10000 Zagreb, Croatia; milan.milosevic@snz.hr; 3 Unit of Obstetrics and Gynecology, Department of Interdisciplinary Medicine (DIM), University of Bari “Aldo Moro”, Policlinico of Bari, Piazza Giulio Cesare 11, 70124 Bari, Italy; 4 School of Medicine, Andrija Štampar School of Public Health, University of Zagreb, 10000 Zagreb, Croatia; 5 Unit of Obstetrics and Gynecology, “Paolo Giaccone” Hospital, 90127 Palermo, Italy; antoniosimone.lagana@unipa.it (A.S.L.); etruscoandrea@gmail.com (A.E.); 6 Department of Health Promotion, Mother and Child Care, Internal Medicine and Medical Specialties (PROMISE), University of Palermo, 90127 Palermo, Italy; vito.chiantera@unipa.it; 7 Unit of Gynecologic Oncology, National Cancer Institute, IRCCS, Fondazione “G. Pascale”, 80131 Naples, Italy

**Keywords:** inherited thrombophilia, healthy antenatal population, perinatal outcome

## Abstract

Background: Despite pregnancy’s hypercoagulable state, the correlation between inherited thrombophilia and thrombotic adverse pregnancy outcomes remains uncertain. The objective of this study was to determine the prevalence of inherited thrombophilic polymorphisms among asymptomatic pregnant individuals and to examine their potential correlation with adverse perinatal outcomes. Methods: in this single-center prospective study, 105 healthy pregnant women were included. Genotyping was conducted for factor V Leiden (FVL), prothrombin gene mutation, methylenetetrahydrofolate reductase enzyme (MTHFR) C677T, MTHFR A1298C, and plasminogen activator inhibitor-1 (PAI-1), alongside the assessment of protein C (PC), protein S (PS), and antithrombin (AT) levels. The study analyzed the association between inherited thrombophilic polymorphisms and pregnancy complications linked to placental insufficiency, such as gestational hypertension (GH), preeclampsia (PE), intrauterine death (IUD), fetal growth restriction (FGR), and placental abruption. Results: The prevalence of identifiable thrombophilic polymorphism mutations was 61.9% (95% confidence interval—CI 52.4–70.8%), with the most common single mutation being PAI-1 4G/5G (12/105, 11.4%, 95% CI 6.4–18.5). The most frequent combined mutation was heterozygosity for MTHFR C677T and PAI-1 (12/105, 11.4%, 95% CI 6.4–18.5). Notably, no FVL homozygous carriers or single homozygous and heterozygous carriers for prothrombin polymorphisms were found. Additionally, no deficiencies in PC and AT were detected among participants. Except for homozygosity for PAI-1, none of the studied polymorphisms demonstrated a significant association with pregnancy complications linked to placental insufficiency. Conclusions: The asymptomatic carriers of inherited thrombophilic polymorphisms do not have an increased risk of adverse perinatal outcomes.

## 1. Introduction

Inherited thrombophilia includes a set of genetic conditions increasing susceptibility to venous thromboembolism (VTE) [1,2]. Despite pregnancy’s innate hypercoagulable state, the potential relationship between inherited thrombophilia and thrombotic adverse pregnancy outcomes, such as fetal growth restriction (FGR), preeclampsia (PE), placental abruption, recurrent pregnancy loss (RPL), and intrauterine death (IUD), remains a topic of ongoing debate [3,4].

Inherited thrombophilias include a range of mutations, notably including the factor V Leiden (FVL) mutation, prothrombin G20210A mutation, and deficiencies of protein C (PC), protein S (PS), and antithrombin (AT), as well as methylenetetrahydrofolate reductase enzyme (MTHFR) 1298 and 677 mutations, and Plasminogen activator inhibitor-1 (PAI-1) mutation [5,6].

Prevalence varies by race, ethnicity, and geographic location [7,8]. In Europe, FVL heterozygosity prevalence ranges from 5% to 8%, with the highest prevalences found in Greece and Sweden, and the mean frequency of the 1691A F5 allele, responsible for factor V Leiden in Europe, was determined to be 3.5% [9], while prothrombin gene mutation prevalence varies from 0.7% to 4%, and is highest in southern Europe [8]. PC deficiency is less common among Caucasians (0.2% to 0.5%) [10], while PS deficiency varies from 0.03% to 0.13% [11]. AT deficiency is rare (0.02% to 0.2%) [12], while MTHFR 677C prevalence ranges from 24% to 64% and MTHFR 1298C from 24% to 46% in Europe [13]. Nearly 50% of Caucasians carry the PAI-1 polymorphism. Distributional differences may arise from diverse genetic and environmental factors globally, with limited data on prevalence in Croatian populations [14].

This study represents the first prospective investigation in Croatia to examine the prevalence and types of thrombophilic gene mutations in healthy pregnant women. The study aimed to gather data on the prevalence of common thrombophilias among healthy pregnant women and to assess any potential associations between various inherited thrombophilic polymorphisms and adverse perinatal outcomes.

## 2. Materials and Methods

This prospective study was conducted at the University Hospital Center Zagreb, Croatia’s tertiary-level maternity hospital, from January 2018 to January 2020. It comprised 105 pregnant women attending the antenatal clinic for routine first-trimester scans, all of whom were locally residing Caucasians. Inclusion criteria were singleton pregnancies with maternal ages between 18 and 37 years, while exclusion criteria comprised multiple pregnancies, pregnancies resulting from in vitro fertilization (IVF), major fetal congenital anomalies, RPL, adverse placental insufficiency-related outcomes in previous pregnancies (such as intrauterine death, fetal growth restriction, and placental abruption), a personal history of thromboembolism, and underlying medical conditions associated with pregnancy complications (e.g., essential hypertension, hypothyroidism, renal disease, or preexisting diabetes).

The study received approval from the Ethical Committee of the School of Medicine, University of Zagreb, and was conducted in compliance with the Declaration of Helsinki. Written informed consent was obtained from all participants.

### 2.1. Laboratory Testing

Following informed consent acquisition, eligible participants underwent genotyping for several polymorphisms, including FVL, prothrombin gene, PAI1, MTHFR ougC677T/A1298C, alongside assessments of their PC, PS, and AT levels. Diagnosis of inherited thrombophilia was considered to be characterized by the presence of one or a combination of seven thrombophilic gene mutations. Genotyping for FVL, prothrombin gene, MTHFR, and PAI1 occurred at the Croatian Institute of Transfusion Medicine, Department of Molecular Diagnostics, while AT and PC values were analyzed at the Department of Platelet and Leukocyte Diagnosis and Hemostasis. PS determination took place at the University Hospital Center Zagreb, Department of Medical Laboratory Diagnostics.

PS, PC, and AT deficiency were defined as plasma activity levels of AT, PC, or PS below the lower limits of the adult reference values (70% for AT, 55% for PC, and 50% for PS), as stated by other publications [15]. PC, PS, and AT levels were evaluated using both clotting-based and chromogenic assays. For FVL, prothrombin gene, PAI1, and MTHFR C677T/A1298C variants assessment, real-time polymerase chain reaction (PCR) techniques were employed.

### 2.2. Pregnancy Management and Data Collection

All participants underwent standard pregnancy surveillance with follow-up examinations in the antenatal care clinic. Fetal surveillance included monthly ultrasound scans until delivery, and all neonates received immediate examination by a pediatrician post-delivery. Data post-delivery, including maternal age, parity, body mass index (BMI), personal and family history of thromboembolism (myocardial infarction, cerebrovascular accident, pulmonary embolism, and deep vein thrombosis), gestational age at delivery, type of delivery, indication for cesarean section, birth weight, and Apgar scores at one and five minutes after birth, were extracted from the hospital’s information system.

Gestational hypertension (GH) was defined as the onset of high blood pressure (≥140/90 mmHg) after 20 weeks of gestation without proteinuria. PE was characterized by high blood pressure and proteinuria (≥0.3 g/24 h urine) or new-onset hypertension with significant end-organ dysfunction, with or without proteinuria, after 20 weeks of gestation or postpartum in previously normotensive women. Placental abruption was diagnosed in cases of vaginal bleeding or concealed hemorrhage after 20 weeks, uterine tenderness, and either fetal distress or maternal shock, or coagulopathy, confirmed by the presence of a blood clot on the maternal side of a delivered placenta. FGR was determined by an estimated fetal weight below the 10th percentile for gestational age, excluding chromosomal abnormalities, congenital malformations, evidence of congenital infection, and substance abuse. IUD was defined as fetal demise after 23 weeks of gestation, excluding chromosomal abnormalities, congenital malformations, and evidence of congenital infection.

Adverse neonatal outcomes were classified into those associated with placental insufficiency (including morbidity linked to FGR, perinatal asphyxia, and neonatal death) and others (such as jaundice, complications due to prematurity, and neonatal infections). Morbidity related to FGR included hypothermia (defined as a newborn’s body temperature being <36.5 °C), hypocalcemia (a total serum calcium concentration of <2 mmol/L in term infants or <1.75 mmol/L in preterm infants), polycythemia (central venous hematocrit of >65% or hemoglobin levels of >22 g/dL), meconium aspiration syndrome (respiratory distress in newborn infants born through meconium-stained amniotic fluid), neonatal pulmonary hypertension (characterized by elevated pulmonary vascular resistance, right-to-left shunting of blood, and hypoxemia), and sepsis. Perinatal asphyxia was diagnosed if at least one of the following criteria was met: a 10 min Apgar score of ≤5, neonatal encephalopathy (evident acute brain injury on MRI indicative of hypoxic-ischemic injury), or an umbilical artery blood pH of ≤7. Neonatal complications attributed to prematurity included anemia (defined as a hemoglobin level of <9 g/dL), jaundice (a total serum bilirubin level of ≥5 mg/dL), infection, respiratory distress syndrome (RDS), intracranial bleeding, persistent ductus arteriosus, necrotizing enterocolitis, and retinopathy.

### 2.3. Data Analysis

We predefined four groups for analysis: a control group (without identifiable polymorphisms), a heterozygous single, a homozygous single, and a combined polymorphism group. The control group comprised individuals with wild-type normal genotypes: PAI1 5G/5G, MTHFR C677C, and MTHFR A1298A. The heterozygous single group included FVL heterozygous, prothrombin heterozygous, PAI1 4G/5G, MTHFR C677T, MTHFR A1298C, PS deficiency (<65 IU/dL), PC deficiency (<65 IU/dL), and AT III deficiency (<75%). The homozygous single group consisted of FVL homozygous, prothrombin homozygous, PAI1 4G/4G, MTHFR T677T, and MTHFR C1298C. The combined polymorphism group comprised all compound mutations observed among participants, such as prothrombin heterozygous + MTHFR C677T, FVL heterozygous + PAI1 4G/5G, and others.

Continuous variables (age, BMI, birthweight, Apgar score, and gestational age) were analyzed using a one-way analysis of variance (ANOVA). The prevalence and types of inherited thrombophilic polymorphisms were expressed as percentages with corresponding 95% confidence intervals (CI). Differences in categorical outcomes were examined using a Fisher–Freeman–Halton’s exact test. A binary logistic regression assessed the association between inherited thrombophilic polymorphisms and pregnancy pathologies related to placental insufficiency. A significance level of 0.05 was applied for all analyses. IBM^®^ Statistical Package for the Social Sciences (SPSS) Statistics version 27.0.1 was utilized for statistical procedures.

## 3. Results

Among the 105 women analyzed, 65 (61.9%, 95% CI 52.4%–70.8%) had identifiable thrombophilic polymorphisms. These subjects were categorized into groups: 40 (38.2%) had no identifiable polymorphisms, 27 (25.7%) were in the heterozygous single group, 9 (8.5%) were in the homozygous single group, and 29 (27.6%) were in the combined polymorphism group. Notably, there were no single homozygous carriers for FVL and no single homozygous or heterozygous carriers for prothrombin polymorphisms. Additionally, no deficiencies in PC or AT were observed among participants.

The most prevalent single mutation was PAI-1 4G/5G heterozygosity (11.4%), while the most common combined mutation was heterozygosity for MTHFR C677T and PAI-1 (11.4%). FVL heterozygosity was observed in two (1.9%) women, and two (1.9%) women exhibited PS deficiency.

In the combined polymorphism group, the predominant mutations were a combination of heterozygosity for MTHFR C677T and PAI-1 (41.38%) and heterozygosity for MTHFR C677T combined with PAI-1 homozygosity (34.48%).

Table 1 presents the prevalence and types of inherited thrombophilia among the cohort of women.

Table 2 outlines the baseline characteristics of the cohort. There were no differences observed among the four groups concerning maternal age, BMI, parity, and family history of VTE.

Table 3 presents the association between four participant groups and perinatal outcomes. Pregnancy complications related to placental insufficiency were observed in six women. Within the control group, one woman (2.5%) developed gestational hypertension (GH). Among heterozygous single carriers, one woman (3.7%) developed GH, and another (3.7%) experienced placental abruption. In the homozygous single group, one patient (11.11%) developed GH, and one (11.11%) was diagnosed with FGR; both were PAI-1 4G/4G homozygous carriers. Among combined polymorphism carriers, two women (6.9%) were antenatally diagnosed with FGR.

No significant differences were observed among the four groups regarding gestational age at delivery, type of delivery, indication for cesarean section (CS), or birthweight. However, a statistically significant difference was noted in Apgar scores; the control group had significantly lower 1st minute Apgar scores, primarily due to two cases of acute hypoxia during labor (*p* = 0.03).

No significant differences were observed among the four groups regarding neonatal outcomes, and among the three groups of inherited thrombophilia carriers, no neonates experienced complications associated with placental insufficiency.

Table 4 illustrates the relationship between individual thrombophilias and pregnancy complications related to placental insufficiency (GH, PE, placental abruption, FGR, and IUD). Except for PAI-1 homozygosity, no other polymorphisms showed significance in predicting pregnancy pathology related to placental insufficiency. Carriers of the PAI-1 polymorphism (4G/4G homozygous) exhibited a significantly higher chance of experiencing pathology related to placental insufficiency (FGR, GH) with an odds ratio (OR) of 12.70 (95% CI: 1.71–93.83; *p* = 0.013). However, due to the small number of participants (N = five), these findings may be coincidental.

## 4. Discussion

Pregnancy is a condition that promotes blood hypercoagulability, becoming more apparent as the pregnancy progresses [16]. The adequate development of placental circulation is crucial for a successful pregnancy, and genetic predisposition to blood clotting disorders can pose a risk for complications related to placental function during pregnancy [17]. The concept of biological hypercoagulability refers to changes in coagulation and fibrinolysis processes that lean toward clot formation during pregnancy. Hypercoagulation leads to increased fibrin turnover, as evidenced by elevated levels of D-Dimers, a sensitive indicator of increased secondary fibrinolytic activation [18]. It is estimated that inherited thrombophilia plays a role in about 50% of the VTE occurrences during pregnancy and the postpartum period [19]. It is widely acknowledged that VTE events during pregnancy, along with inherited thrombophilia, can lead to serious consequences, both maternal and fetal [19]. Anticoagulant therapy appears to be safe and effective for the treatment of pregnancy-related VTE [20], although the prophylactic administration of low molecular weight heparin (LMWH) is not routinely recommended in patients with hereditary thrombophilia without a history of previous VTE or other risk factors, as stated by the majority of guidelines [21,22,23]. Apart from the link to VTE, the connection between hereditary thrombophilias and adverse pregnancy outcomes remains largely unexplored.

Based on our findings, there is no notable association between asymptomatic carriers of inherited thrombophilia and adverse perinatal outcomes, except perhaps for the PAI-1 polymorphism carriers homozygous for 4G/4G. As previously mentioned, in our cohort, only patients with the PAI-1 polymorphism (4G/4G homozygous) showed a significantly higher chance of experiencing pathology related to placental insufficiency (FGR and GH) (OR 12.70; 95% CI: 1.71–93.83; *p* = 0.013). PAI-1, belonging to the serpin superfamily, plays a crucial role in the plasminogen/plasmin system by primarily inhibiting tissue plasminogen activator (tPA) and urokinase-type plasminogen activator (uPA) [24]. Evidence from a recent meta-analysis suggests that the PAI-1 rs1799889 polymorphism might act as a contributing factor to the development of VTE in Caucasians and East Asians, particularly in individuals with deep vein thrombosis (DVT) and those carrying the FVL mutation [25]. Furthermore, it could represent a risk factor for residual venous occlusion after idiopathic DVT [26]. Additionally, the PAI-1 4G/5G polymorphism is significantly involved in the development of RPL by altering metabolic, thrombotic, and immune factors, as shown by multiple studies [27,28,29].

The role of PAI-1 in the pathogenesis of PE and placental insufficiency-related disorders has been extensively investigated by numerous publications in recent years, with conflicting results. PAI-1 could inhibit trophoblast invasion and have a negative impact on the remodeling of maternal uterine spiral arteries, contributing to reproductive diseases such as RPL, PE, IUGR, and even maternal and fetal death [30]. Moreover, increased expression of PAI-1 and tPA, together with decreased levels of AnnexinA2 (ANXA2) were found in placental samples of PE-affected women compared to healthy controls, corroborating these suggestions [31]. Physical activity and certain medications, such as metformin, have the potential to lower PAI-1 levels [29], as for women with polycystic ovarian syndrome (PCOS) [32]. New diagnostic tools, such as non-invasive real-time placental oxygenation measurements through near-infrared spectroscopy (NIRS) could help enhance the current understanding of placental pathophysiology in the development of disorders related to placental insufficiency [33].

A positive association between elevated levels of PAI-1 and increased risk of developing placental disorders has been found by multiple systematic reviews and meta-analyses in the past years [34,35,36]. However, another aggregative analysis, on the contrary, has shown the absence of a statistically significant association between the PAI-1 polymorphism and the development of adverse pregnancy outcomes in asymptomatic nulliparous pregnant patients [37]. The same conclusions were reached by a recent systematic review of 59 studies [38]. Despite finding a positive association between patients with homozygous PAI-1 polymorphism and increased development of placental pathology, we believe that the sample size (N = five) of patients affected by this polymorphism in our cohort is too small to support this hypothesis, and we urge our esteemed colleagues to conduct high-quality research with rigorous patient selection in order to definitively demonstrate or refute this association.

Among our cohort of healthy pregnant carriers, the most prevalent single polymorphisms were PAI-1 mutation and MTHFR, consistent with findings from other European nations. For instance, an Italian case–control study reported a 50.5% prevalence of PAI-1 gene 4G/5G polymorphisms among Italians [39], while, in a study from Jordan, the prevalence was estimated to be around 66% [40]. Wilcken et al. noted an increasing prevalence of the MTHFR 6777 TT genotype in Europe, with higher rates observed in southern regions [41]. A recent study from Romania found a prevalence of MTHFR polymorphisms of 38.2% for C677T and of 40.3% for MTHFR A1298C in their population [42]. In our antenatal population, 3.8% were homozygous carriers of MTHFR C677T, 5.7% were heterozygous carriers, and 4.8% exhibited heterozygosity for MTHFR A1298C; these results were slightly lower than the rates reported in other Mediterranean countries. Notably, no women in our study were homozygous for MTHFR A1298C. Additionally, twenty-seven women were identified as combined carriers of MTHFR and PAI-1 polymorphisms, with the majority (44.4%) being MTHFR C677T and PAI-1 4G/5G heterozygous, aligning with prevalence rates observed in other Caucasian populations [7,8,9] and with a recent retrospective analysis [43].

Variations in the prevalence of FVL and prothrombin gene mutation across European regions likely stem from historical population migrations. In Central Europe, FVL heterozygosity prevalence is 4%, while in Western Europe, it rises to 7.8%, with prothrombin gene heterozygosity at 3.5% [9]. Geographic disparities in FVL incidence along the north–south axis have been noted. In our study, FVL prevalence was 1.9%, with no instances of isolated prothrombin gene polymorphism; prothrombin gene mutations were only observed in combination with MTHFR C677T heterozygosity (1%).

The prevalence of PS deficiency is estimated at less than 0.5% in the general European population [10,11]. In our study, PS deficiency was observed in 1.9% of participants. Overall, our findings suggest that the prevalence of prothrombin gene, PAI-1, MTHFR 1298 and MTHFR 677 polymorphisms, and PS deficiency in healthy pregnant women in Croatia does not significantly deviate from that observed in neighboring European countries. However, FVL prevalence appears slightly lower in Croatia.

Hereditary thrombophilia can significantly negatively impact female patients of reproductive age, starting from the periconceptional period, increasing the risk of RPL [44,45], like other pathological conditions such as uterine aging or Müllerian anomalies [46,47]. Furthermore, it is crucial to conduct screenings for women with hereditary thrombophilias and to maintain a high level of suspicion for VTE during pregnancy [48]. This proactive approach ensures early detection and appropriate management, safeguarding both the maternal and fetal health throughout the gestational period. Unfortunately, no biomarker has demonstrated adequate sensitivity and specificity in the early identification of VTE during pregnancy, as shown by a recent observational cohort study [49].

Another important aspect concerns the management of labor in patients with hereditary thrombophilia. Controversies persist regarding the choice between inducing labor and awaiting spontaneous labor, as a long interval without anticoagulation is a thrombosis risk factor, while a short interval increases the risks of delivering without epidural analgesia or postpartum hemorrhage [50]. On the other hand, premature induction or induction without proper cervical ripening can lead to an increased rate of induction failure, number of misoprostol doses, longer time of induction, and higher cesarean section and episiotomy rate, as in obese and older women [51,52].

Numerous studies have explored the link between inherited thrombophilias and adverse pregnancy outcomes, yielding conflicting findings [53,54]. Thrombophilia-induced clotting disorders may partly result from thrombosis in the spiral artery and intervillous spaces of the maternal placenta, as well as inhibition of extra-villous trophoblast differentiation, avoiding essential placental tissue perfusion for fetal growth. Meta-analyses of case–control studies consistently highlight associations between inherited thrombophilias and late pregnancy complications like IUD, FGR, placental abruption, and PE [55,56]. On this regard, a Serbian study demonstrated better perinatal outcomes in women with hereditary thrombophilias treated with prophylactic LMWH, compared to untreated women [57], consistent with another research study [58]. There is a need for more evidence on the actual utility of prophylactic anticoagulant therapy in patients with asymptomatic hereditary thrombophilia and no prior history of VTE, as anticoagulant therapy carries bleeding risks and may complicate the outcome of surgical procedures that may sometimes become necessary urgently in pregnant women, such as in the case of symptomatic fibroids or ectopic pregnancies [59,60]. However, recent prospective cohort studies have shown that women with thrombophilias do not exhibit increased risks of adverse pregnancy outcomes, consistent with our findings [61,62].

### Strengths and Limitations

Our single-center prospective study indicates that asymptomatic carriers of inherited thrombophilic polymorphisms are unlikely to face pathology associated with placental insufficiency. While isolated cases of GH, placental abruption, and FGR were identified across all three groups, the small number of cases within each category does not suggest clinically significant disparities, except, perhaps, for carriers of the PAI-1 polymorphism (4G/4G homozygous). However, these findings warrant caution due to the small patient sample size.

One limitation of our study is the relatively small participant size, but it is unlikely that a larger sample would reveal clinically significant disparities. Another limitation of our research is that the levels of protein C, protein S, and antithrombin were determined only during pregnancy, as the activity levels could be influenced by the pregnant state.

A key discovery of this study is the confirmation that the majority of asymptomatic carriers of inherited thrombophilia can expect uncomplicated pregnancies, which is pertinent in treatment considerations for these women. Nonetheless, it is important to note that thrombophilic patients with a history of DVT, or obstetric complications related to placental pathology, may represent a distinct phenotype with potentially higher complication rates compared to asymptomatic carriers.

## 5. Conclusions

To conclude, our findings suggest that the prevalence and types of common inherited thrombophilic polymorphisms in the Croatian antenatal population align with those observed in other Caucasian populations. Moreover, our study does not indicate a significant association between asymptomatic carriers of inherited thrombophilia and pathology linked to placental insufficiency.

## Figures and Tables

**Table 1:** Prevalence and types of inherited thrombophilic polymorphisms among low-risk pregnant women (N = 105).

	N	%	95% CI	
			Lower	Upper
FVL heterozygous	2	1.9%	0.4%	6.0%
Protein S deficiency ^†^	2	1.9%	0.4%	6.0%
PAI1 polymorphism				
4G/5G heterozygous	12	11.4%	6.4%	18.5%
4G/4G homozygous	5	4.8%	1.8%	10.1%
MTHFR A1298C polymorphism				
A1298C heterozygous	5	4.8%	1.8%	10.1%
MTHFR C677T polymorphism				
C677T heterozygous	6	5.7%	2.4%	11.4%
C677T homozygous	4	3.8%	1.3%	8.8%
Combined polymorphisms				
Prothrombin heterozygous, MTHFR C677T heterozygous	1	1.0%	0.1%	4.4%
FVL heterozygous, PAI1 4G/5G heterozygous	1	1.0%	0.1%	4.4%
MTHFR C677T heterozygous, PAI1 4G/4G homozygous	10	9.5%	5.0%	16.2%
MTHFR C677T heterozygous, PAI1 4G/5G heterozygous	12	11.4%	6.4%	18.5%
MTHFR C677T homozygous, PAI1 4G/5G heterozygous	3	2.9%	0.8%	7.4%
MTHFR C677T homozygous, PAI1 4G/4G homozygous	2	1.9%	0.4%	6.0%

^†^ Protein S (PS) deficiency defined as PS plasma activity levels of <50%.

**Table 2:** Demographics.

Variable	Control Group	Heterozygous Single	Homozygous Single	Combined Polymorphisms	*p*
	N = 40	N = 27	N = 9	N = 29	
Age (mean ± S.D.)	31.4 ± 4.1	31.8 ± 2.3	31.1 ± 2.6	31.8 ± 3.7	0.731
BMI (mean ± S.D.)	25.6 ± 3.5	25.6 ± 4.7	24.7 ± 3.5	23.6 ± 2.5	0.142
Parity (N, %)					0.192
0	24 (60)	14 (51.9)	4 (44.6)	23 (79.3)	
1	9 (22.5)	10 (37)	3 (33.3)	3 (10.3)	
>2	7 (17.5)	3 (11.1)	2 (22.2)	3 (10.3)	
Family history for VTE ^†^ (N, %)					0.541
Negative	36 (90)	22 (81.5)	7 (77.8)	23 (79.3)	
Positive	4 (10)	5 (18.5)	2 (22.2)	6 (20.7)	

Abbreviations: BMI—body mass index; N—number; TE—thromboembolic diseases. ^†^ Cerebrovascular insult, deep vein thrombosis, pulmonary embolism, myocardial infraction.

**Table 3:** Pregnancy complications and perinatal outcomes.

Variable	Control Group	Heterozygous Single	Homozygous Single	Combined Polymorphisms	*p*
	N = 40	N = 27	N = 9	N = 29	
Pathology of pregnancy (N, %)					0.067
Gestational hypertension	1 (2.5)	1 (3.7)	1 (11.1)	0	
Placental abruption	0	1 (3.7)	0	0	
Fetal growth restriction	0	0	1 (11.1)	2 (6.9)	
Gestational age at delivery (mean ± S.D.)	38.9 ± 1.6	39.2 ± 0.1	39.1 ± 1.3	39.4 ± 1.1	0.634
Caesarean section (N, %)	9 (22.5)	6 (22.2)	1 (11.1)	11 (37.9)	0.356
Due to complications related to placental insufficiency *	0	1	0	1	0.610
Abnormal CTG in labor ^†^	2	2	0	1	
Other ^‡^	7	3	1	9	
Birthweight (mean ± S.D.)	3441 ± 536	3521 ± 398	3231 ± 498	3402 ± 392	0.523
Apgar score (mean ± S.D.)					
1st minute	9.55 ± 1.1	10.00 ± 0.0	9.89 ± 0.3	10.00 ± 0.0	0.033
5th minute	9.83 ± 0.8	10.00 ± 0.0	9.89 ± 0.3	10.00 ± 0.0	0.222
Neonatal outcome (N, %)					
Without complications related to antenatal placental insufficiency *	36 (90)	27 (100)	9 (100)	26 (89.6)	0.519
Perinatal asphyxia ^§^	1 (2.5)	0	0	0	
Other (jaundice, complications due to prematurity ^II^)	3 (7.5)	0	0	3 (10.3)	

* Gestational hypertension, preeclampsia, fetal growth restriction, preeclampsia, placental abruption, intrauterine fetal death. ^†^ Variable decelerations, late decelerations, prolonged decelerations. ^‡^ Fetal malpresentation presentation, cephalopelvic disproportion, placenta previa, suspected fetal macrosomia, etc. ^§^ 10 min Apgar Score ≤ 5, neonatal encephalopathy, umbilical artery blood pH ≤ 7. ^II^ Anemia, jaundice, infection, respiratory distress syndrome, intracranial bleeding, patent ductus arteriosus, necrotizing enterocolitis, retinopathy.

**Table 4:** The association between inherited thrombophilic polymorphisms and pregnancy complications related to placental insufficiency: binary logistic regression.

	Pregnancy Complications		OR	95% CI	*p*
	No	Yes			
	N = 98 (%)	N = 7 (%)			
FVL heterozygous	2 (2)	0	NA		
Protein S deficiency ^†^	2 (2)	0	NA		
PAI1 polymorphism					
4G/5G heterozygous	12 (12.2)	0	NA		
4G/4G homozygous	3 (3.1)	2 (28.6)	12.7	1.7–93.8	0.013
MTHFR A1298C polymorphism					
A1298C heterozygous	4 (4.1)	1 (14.3)	3.9	0.3–40.7	0.253
MTHFR C677T polymorphism					
C677T heterozygous	5 (5.1)	1 (14.3)	3.1	0.3–30.9	0.335
C677T homozygous	4 (4.1)	0	NA		
Combined polymorphisms					
Prothrombin heterozygous, MTHFR C677T heterozygous	1 (1)	0	NA		
FVL heterozygous, PAI1 4G/5G heterozygous	1 (1)	0	NA		
MTHFR C677T heterozygous, PAI1 4G/4G homozygous	8 (8.2)	2	4.5	0.7–27.1	0.100
MTHFR C677T heterozygous, PAI1 4G/5G heterozygous	12 (12.2)	0	NA		
MTHFR C677T homozygous, PAI1 4G/5G heterozygous	3 (3.1)	0	NA		
MTHFR C677T homozygous, PAI1 4G/4G homozygous	2 (2)	0	NA		

Abbreviations: CI—confidence interval; N—number; NA—not applicable. ^†^ Protein S (PS) deficiency defined as PS plasma activity levels <50%.

## Data Availability

Due to privacy considerations, the data for this study are not publicly available. However, they could be provided upon request from the authors.

## References

[1] Dautaj A., Krasi G., Bushati V., Precone V., Gheza M., Fioretti F., Sartori M., Costantini A., Benedetti S., Bertelli M. (2019). Hereditary Thrombophilia. Acta Biomed..

[2] Dicks A.B., Moussallem E., Stanbro M., Walls J., Gandhi S., Gray B.H. (2024). A Comprehensive Review of Risk Factors and Thrombophilia Evaluation in Venous Thromboembolism. J. Clin. Med..

[3] Bezgin T., Kaymaz C., Akbal Ö., Yılmaz F., Tokgöz H.C., Özdemir N. (2018). Thrombophilic Gene Mutations in Relation to Different Manifestations of Venous Thromboembolism: A Single Tertiary Center Study. Clin. Appl. Thromb. Hemost..

[4] O’Rourke E., Faryal R., Blondon M., Middeldorp S., Ní Áinle F. (2024). VTE Risk Assessment and Prevention in Pregnancy. Hamostaseologie.

[5] Stevens S.M., Woller S.C., Bauer K.A., Kasthuri R., Cushman M., Streiff M., Lim W., Douketis J.D. (2016). Guidance for the Evaluation and Treatment of Hereditary and Acquired Thrombophilia. J. Thromb. Thrombolysis.

[6] Khider L., Gendron N., Mauge L. (2022). Inherited Thrombophilia in the Era of Direct Oral Anticoagulants. Int. J. Mol. Sci..

[7] Yıldız E., Türkmen F.M. (2020). Factor V Leiden Mutation Frequency and Geographical Distribution in Turkish Population. J. Transl. Int. Med..

[8] Jadaon M.M. (2011). Epidemiology of Prothrombin G20210A Mutation in the Mediterranean Region. Mediterr. J. Hematol. Infect. Dis..

[9] Clark J.S.C., Adler G., Salkic N.N., Ciechanowicz A. (2013). Allele Frequency Distribution of 1691G>A F5 (Which Confers Factor V Leiden) across Europe, Including Slavic Populations. J. Appl. Genet..

[10] Vrtel P., Slavik L., Vodicka R., Stellmachova J., Prochazka M., Prochazkova J., Ulehlova J., Rohon P., Simurda T., Stasko J. (2022). Detection of Unknown and Rare Pathogenic Variants in Antithrombin, Protein C and Protein S Deficiency Using High-Throughput Targeted Sequencing. Diagnostics.

[11] Wypasek E., Corral J., Alhenc-Gelas M., Sydor W., Iwaniec T., Celińska-Lowenhoff M., Potaczek D.P., Blecharczyk A., Zawilska K., Musiał J. (2017). Genetic Characterization of Antithrombin, Protein C, and Protein S Deficiencies in Polish Patients. Pol. Arch. Intern. Med..

[12] Kumar R., Chan A.K.C., Dawson J.E., Forman-Kay J.D., Kahr W.H.A., Williams S. (2014). Clinical Presentation and Molecular Basis of Congenital Antithrombin Deficiency in Children: A Cohort Study. Br. J. Haematol..

[13] Yang B., Liu Y., Li Y., Fan S., Zhi X., Lu X., Wang D., Zheng Q., Wang Y., Wang Y. (2013). Geographical Distribution of *MTHFR* C677T, A1298C and *MTRR* A66G Gene Polymorphisms in China: Findings from 15357 Adults of Han Nationality. PLoS ONE.

[14] Lovricević I., Franjić B.D., Tomicić M., Vrkić N., De Syo D., Hudorović N., Sonicki Z., Loncar R. (2004). 5, 10-Methylenetetrahydrofolate Reductase (MTHFR) 677 C -> T Genetic Polymorphism in 228 Croatian Volunteers. Coll. Antropol..

[15] Kobayashi T., Sugiura K., Ojima T., Serizawa M., Hirai K., Morishita E. (2024). Thrombosis-Related Characteristics of Pregnant Women with Antithrombin Deficiency, Protein C Deficiency and Protein S Deficiency in Japan. Thromb. J..

[16] Bhave A.A. (2019). Coagulopathies in Pregnancy: What an Obstetrician Ought to Know!. J. Obstet. Gynaecol. India.

[17] Campello E., Spiezia L., Adamo A., Simioni P. (2019). Thrombophilia, Risk Factors and Prevention. Expert Rev. Hematol..

[18] Siennicka A., Kłysz M., Chełstowski K., Tabaczniuk A., Marcinowska Z., Tarnowska P., Kulesza J., Torbe A., Jastrzębska M. (2020). Reference Values of D-Dimers and Fibrinogen in the Course of Physiological Pregnancy: The Potential Impact of Selected Risk Factors-A Pilot Study. Biomed. Res. Int..

[19] Daughety M.M., Samuelson Bannow B.T., DeLoughery T.G. (2019). Hemostasis and Thrombosis in Pregnancy. Hemostasis and Thrombosis.

[20] Romualdi E., Dentali F., Rancan E., Squizzato A., Steidl L., Middeldorp S., Ageno W. (2013). Anticoagulant Therapy for Venous Thromboembolism during Pregnancy: A Systematic Review and a Meta-Analysis of the Literature. J. Thromb. Haemost..

[21] Hart C., Bauersachs R., Scholz U., Zotz R., Bergmann F., Rott H., Linnemann B. (2020). Prevention of Venous Thromboembolism during Pregnancy and the Puerperium with a Special Focus on Women with Hereditary Thrombophilia or Prior VTE-Position Paper of the Working Group in Women’s Health of the Society of Thrombosis and Haemostasis (GTH). Hamostaseologie.

[22] Ormesher L., Simcox L., Tower C., Greer I.A. (2016). Management of Inherited Thrombophilia in Pregnancy. Womens Health.

[23] Reducing the Risk of Thrombosis and Embolism during Pregnancy and the Puerperium (Green-Top Guideline No. 37a). https://www.rcog.org.uk/guidance/browse-all-guidance/green-top-guidelines/reducing-the-risk-of-thrombosis-and-embolism-during-pregnancy-and-the-puerperium-green-top-guideline-no-37a/.

[24] Sillen M., Declerck P.J. (2020). Targeting PAI-1 in Cardiovascular Disease: Structural Insights into PAI-1 Functionality and Inhibition. Front. Cardiovasc. Med..

[25] Huang G., Wang P., Li T., Deng X. (2019). Genetic Association between Plasminogen Activator Inhibitor-1 Rs1799889 Polymorphism and Venous Thromboembolism: Evidence from a Comprehensive Meta-analysis. Clin. Cardiol..

[26] Li W., Cao S., Liu B., Zhang Z., Liu Z., Feng H. (2023). Influence of the 4G/5G Polymorphism of Plasminogen Activator Inhibitor-1 Gene in Acute Unprovoked Deep Vein Thrombosis and Residual Vein Thrombosis. J. Vasc. Surg. Venous Lymphat. Disord..

[27] Cernera G., Liguori R., Bruzzese D., Castaldo G., De Placido G., Conforti A., Amato F., Alviggi C., Comegna M. (2024). The Relevance of Prothrombotic Genetic Variants in Women Who Experienced Pregnancy Loss or Embryo Implantation Failure: A Retrospective Analysis of 1922 Cases. Int. J. Gynaecol. Obstet..

[28] Wen Y., He H., Zhao K. (2023). Thrombophilic Gene Polymorphisms and Recurrent Pregnancy Loss: A Systematic Review and Meta-Analysis. J. Assist. Reprod. Genet..

[29] Zhai J., Li Z., Zhou Y., Yang X. (2022). The Role of Plasminogen Activator Inhibitor-1 in Gynecological and Obstetrical Diseases: An Update Review. J. Reprod. Immunol..

[30] Ye Y., Vattai A., Zhang X., Zhu J., Thaler C.J., Mahner S., Jeschke U., von Schönfeldt V. (2017). Role of Plasminogen Activator Inhibitor Type 1 in Pathologies of Female Reproductive Diseases. Int. J. Mol. Sci..

[31] Ruikar K., Khode V., Shetty S.S., Sarathkumar E., Patil P., Patil S., Bargale A., Sadashiv R., Shetty P. (2023). Association of Pro-Fibrinolytic Receptor AnnexinA2 with Tissue Plasminogen Activator/Inhibitor-1 in Pre-Eclampsia. Afr. Health Sci..

[32] Etrusco A., Mikuš M., D’Amato A., Barra F., Planinić P., Goluža T., Buzzaccarini G., Marušić J., Tešanović M., Laganà A.S. (2024). Incretin Hormone Secretion in Women with Polycystic Ovary Syndrome: Roles of Obesity, Insulin Sensitivity and Treatment with Metformin and GLP-1s. Biomedicines.

[33] Jakovac M.B., Etrusco A., Mikuš M., Roje D., Marusic J., Palada I., Kosovic I., Aracic N., Sunj M., Laganà A.S. (2023). Evaluation of Placental Oxygenation by Near-Infrared Spectroscopy in Relation to Ultrasound Maturation Grade in Physiological Term Pregnancies. Open Med..

[34] Zhao L., Bracken M.B., Dewan A.T., Chen S. (2013). Association between the SERPINE1 (PAI-1) 4G/5G Insertion/Deletion Promoter Polymorphism (Rs1799889) and Pre-Eclampsia: A Systematic Review and Meta-Analysis. Mol. Hum. Reprod..

[35] Morgan J.A., Bombell S., McGuire W. (2013). Association of Plasminogen Activator Inhibitor-Type 1 (-675 4G/5G) Polymorphism with Pre-Eclampsia: Systematic Review. PLoS ONE.

[36] Giannakou K., Evangelou E., Papatheodorou S.I. (2018). Genetic and Non-Genetic Risk Factors for Pre-Eclampsia: Umbrella Review of Systematic Reviews and Meta-Analyses of Observational Studies. Ultrasound Obstet. Gynecol..

[37] Said J.M., Tsui R., Borg A.J., Higgins J.R., Moses E.K., Walker S.P., Monagle P.T., Brennecke S.P. (2012). The PAI-1 4G/5G Polymorphism Is Not Associated with an Increased Risk of Adverse Pregnancy Outcome in Asymptomatic Nulliparous Women. J. Thromb. Haemost..

[38] Agersnap I., Nissen P.H., Hvas A.-M. (2022). The Role of Plasminogen Activator Inhibitor Type 1 (PAI-1) in Placenta-Mediated Pregnancy Complications: A Systematic Review. Semin. Thromb. Hemost..

[39] Burzotta F., Di Castelnuovo A., Amore C., D’Orazio A., Di Bitondo R., Donati M.B., Iacoviello L. (1998). 4G/5G Promoter PAI-1 Gene Polymorphism Is Associated with Plasmatic PAI-1 Activity in Italians: A Model of Gene-Environment Interaction. Thromb. Haemost..

[40] Al-Zoubi N., Alrabadi N., Kheirallah K., Alqudah A. (2021). Prevalence and Multiplicity of Thrombophilia Genetic Polymorphisms of *FV*, *MTHFR*, *FII*, and *PAI-I*: A Cross-Sectional Study on a Healthy Jordanian Population. Int. J. Gen. Med..

[41] Wilcken B., Bamforth F., Li Z., Zhu H., Ritvanen A., Renlund M., Stoll C., Alembik Y., Dott B., Czeizel A.E. (2003). Geographical and Ethnic Variation of the 677C>T Allele of 5,10 Methylenetetrahydrofolate Reductase (MTHFR): Findings from over 7000 Newborns from 16 Areas World Wide. J. Med. Genet..

[42] Chita D.S., Tudor A., Christodorescu R., Buleu F.N., Sosdean R., Deme S.M., Mercea S., Pop Moldovan A., Pah A.M., Docu Axelerad A. (2020). *MTHFR* Gene Polymorphisms Prevalence and Cardiovascular Risk Factors Involved in Cardioembolic Stroke Type and Severity. Brain Sci..

[43] Donmez H.G., Beksac M.S. (2023). Association of Single Nucleotide Polymorphisms (4G/5G) of Plasminogen Activator Inhibitor-1 and the Risk Factors for Placenta-Related Obstetric Complications. Blood Coagul. Fibrinolysis.

[44] Samfireag M., Potre C., Potre O., Tudor R., Hoinoiu T., Anghel A. (2022). Approach to Thrombophilia in Pregnancy—A Narrative Review. Medicina.

[45] Ahangari N., Doosti M., Mousavifar N., Attaran M., Shahrokhzadeh S., Memarpour S., Ghayoor Karimiani E. (2019). Hereditary Thrombophilia Genetic Variants in Recurrent Pregnancy Loss. Arch. Gynecol. Obstet..

[46] Tinelli A., Andjić M., Morciano A., Pecorella G., Malvasi A., D’Amato A., Sparić R. (2024). Uterine Aging and Reproduction: Dealing with a Puzzle Biologic Topic. Int. J. Mol. Sci..

[47] Mraihi F., Buzzaccarini G., D’Amato A., Laganà A.S., Basly J., Mejri C., Hafsi M., Chelli D., Ghali Z., Bianco B. (2024). Cornual Pregnancy: Results of a Single-Center Retrospective Experience and Systematic Review on Reproductive Outcomes. Medicina.

[48] Colucci G., Tsakiris D.A. (2020). Thrombophilia Screening Revisited: An Issue of Personalized Medicine. J. Thromb. Thrombolysis.

[49] Hunt B.J., Parmar K., Horspool K., Shephard N., Nelson-Piercy C., Goodacre S., on behalf of the DiPEP research group (2018). The DiPEP (Diagnosis of PE in Pregnancy) Biomarker Study: An Observational Cohort Study Augmented with Additional Cases to Determine the Diagnostic Utility of Biomarkers for Suspected Venous Thromboembolism during Pregnancy and Puerperium. Br. J. Haematol..

[50] Mauny L., Peyronnet V., Peynaud-Debayle E., Picone O., Nebout S., Mandelbrot L. (2023). Induction or Spontaneous Labor for Pregnant Patients on Anticoagulants?. J. Gynecol. Obstet. Human. Reprod..

[51] Sfregola G., Sfregola P., Ruta F., Zendoli F., Musicco A., Garzon S., Uccella S., Etrusco A., Chiantera V., Terzic S. (2023). Effect of Maternal Age and Body Mass Index on Induction of Labor with Oral Misoprostol for Premature Rupture of Membrane at Term: A Retrospective Cross-Sectional Study. Open Med..

[52] Etrusco A., Sfregola G., Zendoli F., Musicco A., Belpiede A., Della Pietà C., Giannini A., Mikuš M., Venezia R., Garzon S. (2024). Effect of Maternal Age and BMI on Induction of Labor Using Oral Misoprostol in Late-Term Pregnancies: A Retrospective Cross-Sectional Study. Gynecol. Obstet. Invest..

[53] Voicu D.I., Munteanu O., Gherghiceanu F., Arsene L.V., Bohiltea R.E., Gradinaru D.M., Cirstoiu M.M. (2020). Maternal Inherited Thrombophilia and Pregnancy Outcomes. Exp. Ther. Med..

[54] Dłuski D., Mierzyński R., Poniedziałek-Czajkowska E., Leszczyńska-Gorzelak B. (2018). Adverse Pregnancy Outcomes and Inherited Thrombophilia. J. Perinat. Med..

[55] Chen Y., Wang T., Liu X., Ye C., Xing D., Wu R., Li F., Chen L. (2022). Low Molecular Weight Heparin and Pregnancy Outcomes in Women with Inherited Thrombophilia: A Systematic Review and Meta-Analysis. J. Obstet. Gynaecol. Res..

[56] Liu X., Chen Y., Ye C., Xing D., Wu R., Li F., Chen L., Wang T. (2021). Hereditary Thrombophilia and Recurrent Pregnancy Loss: A Systematic Review and Meta-Analysis. Hum. Reprod..

[57] Dugalic S., Petronijevic M., Stefanovic A., Stefanovic K., Petronijevic S.V., Stanisavljevic D., Kepeci S.P., Milincic N., Pantic I., Perovic M. (2019). Comparison of 2 Approaches in Management of Pregnant Women with Inherited Trombophilias: Prospective Analytical Cohort Study. Medicine.

[58] Aracic N., Roje D., Jakus I.A., Bakotin M., Stefanovic V. (2016). The Impact of Inherited Thrombophilia Types and Low Molecular Weight Heparin Treatment on Pregnancy Complications in Women with Previous Adverse Outcome. Yonsei Med. J..

[59] Babunashvili E.L., Son D.Y., Buyanova S.N., Schukina N.A., Popov A.A., Chechneva M.A., Glebov T.A., D’Amato A., Haydamous J., Chiantera V. (2023). Outcomes of Laparotomic Myomectomy during Pregnancy for Symptomatic Uterine Fibroids: A Prospective Cohort Study. J. Clin. Med..

[60] Cucinella G., Gullo G., Etrusco A., Dolce E., Culmone S., Buzzaccarini G. (2021). Early Diagnosis and Surgical Management of Heterotopic Pregnancy Allows Us to Save the Intrauterine Pregnancy. Prz. Menopauzalny.

[61] Fernández Arias M., Mazarico E., Gonzalez A., Muniesa M., Molinet C., Almeida L., Gómez Roig M.D. (2019). Genetic Risk Assessment of Thrombophilia in Patients with Adverse Obstetric Outcomes. PLoS ONE.

[62] Freedman A.A., Hogue C.J., Dudley D.J., Silver R.M., Stoll B.J., Pinar H., Goldenberg R.L., Drews-Botsch C. (2017). Associations between Maternal and Fetal Inherited Thrombophilias, Placental Characteristics Associated with Vascular Malperfusion, and Fetal Growth. TH Open.

